# Informed-Learning-Guided Visual Question Answering Model of Crop Disease

**DOI:** 10.34133/plantphenomics.0277

**Published:** 2024-12-16

**Authors:** Yunpeng Zhao, Shansong Wang, Qingtian Zeng, Weijian Ni, Hua Duan, Nengfu Xie, Fengjin Xiao

**Affiliations:** ^1^ College of Computer Science and Engineering, Shandong University of Science and Technology, Qingdao 266590, China.; ^2^ Agricultural Information Institute of CAAS, Beijing 100081, China.; ^3^ National Climate Center, Beijing 100081, China.

## Abstract

In contemporary agriculture, experts develop preventative and remedial strategies for various disease stages in diverse crops. Decision-making regarding the stages of disease occurrence exceeds the capabilities of single-image tasks, such as image classification and object detection. Consequently, research now focuses on training visual question answering (VQA) models. However, existing studies concentrate on identifying disease species rather than formulating questions that encompass crucial multiattributes. Additionally, model performance is susceptible to the model structure and dataset biases. To address these challenges, we construct the informed-learning-guided VQA model of crop disease (ILCD). ILCD improves model performance by integrating coattention, a multimodal fusion model (MUTAN), and a bias-balancing (BiBa) strategy. To facilitate the investigation of various visual attributes of crop diseases and the determination of disease occurrence stages, we construct a new VQA dataset called the Crop Disease Multi-attribute VQA with Prior Knowledge (CDwPK-VQA). This dataset contains comprehensive information on various visual attributes such as shape, size, status, and color. We expand the dataset by integrating prior knowledge into CDwPK-VQA to address performance challenges. Comparative experiments are conducted by ILCD on the VQA-v2, VQA-CP v2, and CDwPK-VQA datasets, achieving accuracies of 68.90%, 49.75%, and 86.06%, respectively. Ablation experiments are conducted on CDwPK-VQA to evaluate the effectiveness of various modules, including coattention, MUTAN, and BiBa. These experiments demonstrate that ILCD exhibits the highest level of accuracy, performance, and value in the field of agriculture. The source codes can be accessed at https://github.com/SdustZYP/ILCD-master/tree/main.

## Introduction

The Food and Agriculture Organization of the United Nations has reports that diseases are responsible for causing annual global crop losses of 10% to 30% [1]. This highlights the importance of implementing timely preventive measures. In the event of crop diseases, experts conduct on-site inspections and offer solutions. Experts follow a diagnostic process that progresses from general observation to comprehensive analysis, divided into 3 steps: (a) identifying the specific part of the crop (such as fruit), (b) assessing local abnormalities (such as fruit rot), and (c) evaluating the characteristics of disease spots (such as color, shape, and number). This process is time-consuming and labor-intensive [2] Consequently, the situation has resulted in a growing focus on automating the identification of crop diseases through the utilization of machine learning methods [3,4].

Advancements in computer vision and natural language processing lead to the widespread use of deep learning in the detection of crop diseases. However, current models primarily rely on unimodal data (such as images or spectral data) for the identification of diseases [5–7]. These models are highly effective in classifying disease images. However, these models have limitations in addressing specific user questions about image content. According to information theory, features with higher information are more valuable to the classifier [8]. Consequently, recent studies integrate multimodal data into crop disease detection models to enhance accuracy. For instance, (a) Zhou et al. [9] effectively identified common tomato and cucumber diseases by combined learning from image–text correlations. (b) Zhang et al. [10] combined image data and environmental factors in a multimodal model to classify tomato diseases. (c) Patil and Kumar [11] also utilized sensor and camera data to classify rice diseases, surpassing unimodal models. These research studies suggest that multimodal fusion improves the extraction of intricate features, highlighting the necessity for additional investigation of multimodal methods in crop disease detection [12–14]. Furthermore, enhancing performance requires the integration of combined images with environmental or meteorological data, as well as the generation of more informative and comprehensible descriptions.

**Fig. 1.**
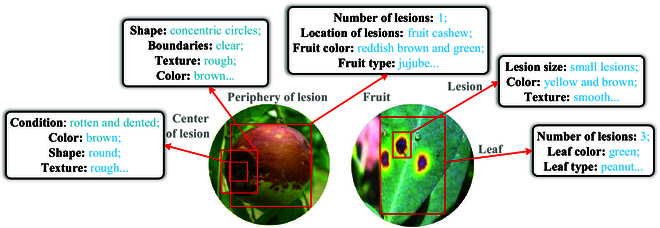
Attribute information is contained in Crop Disease Multi-attribute Visual Question Answering with Prior Knowledge (CDwPK-VQA). Consistent with the task specifications, we define the aforementioned attributes in CDwPK-VQA, which include characteristics such as color, shape, size, disease type, and crop type.

Existing models for classifying crop diseases are unable to provide detailed textual descriptions of visual features. The descriptions that visually characterize diseases are crucial for accurately identifying stages of the diseases [15,16]. To address this challenge, we construct a new dataset for crop diseases that includes multiple attributes for visual question answering (VQA). This dataset is annotated in collaboration with agricultural experts and involves 19 common diseases with detailed visual attributes (Fig. 1). By utilizing this dataset, the VQA model is capable of learning the intricate relationships between images and questions and allowing for inference across various visual attributes. However, the size of the dataset is limited, and the training of the VQA task depends on the ability to understand a diverse range of images and text [17,18]. Consequently, we incorporate prior knowledge of crop diseases into the dataset to enhance model convergence and accuracy.

**Fig. 2.**
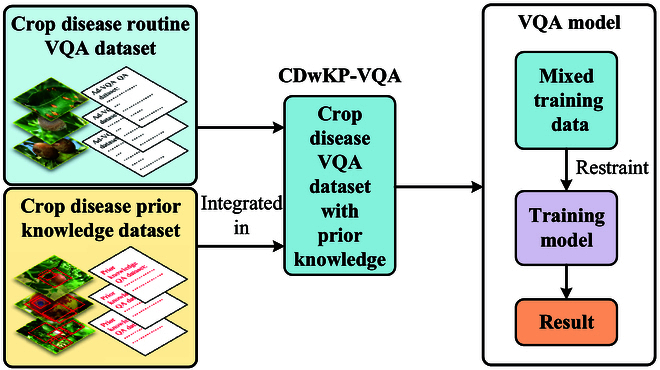
The concept of informed learning. Integrated into the visual question answering (VQA) model, CDwPK-VQA constitutes a fusion of 2 information sources. The crop disease prior knowledge dataset includes images that are annotated with object regions and the corresponding question–answer pairs.

Increasing evidence suggests that artificial intelligence models trained on smaller datasets face marked performance risks [19,20]. Purely data-driven models necessitate large labeled datasets and, consequently, have lower performance when model training data are insufficient [21]; in other words, if there are not enough training data, the model may struggle to meet the work requirements. To address these limitations, advancements occur in various disciplines. (a) In the field of fluid mechanics, the use of trainable spectral filters has contributed to the improvement of turbulence prediction models [22]. (b) In condensed matter physics, the application of graph neural networks leads to advancements in electronic structure predictions [23]. The enhancement of model performance methods also involved the incorporation of natural law constraints during training [22] and the utilization of large datasets [23]. In order to decrease the labor and time requirements of dataset labeling and enhance model performance, we integrated prior knowledge of crop diseases into Crop Disease Multi-attribute VQA with Prior Knowledge (CDwPK-VQA). As shown in Fig. 2, informed learning combines mixed information sources to improve the performance of the VQA model.

This research presents the informed-learning-guided VQA model of crop disease (ILCD). ILCD provides a solution for conducting multiattribute inquiries related to crop disease images. The model incorporates coattention mechanisms, multimodal feature fusion (MUTAN), and bias-balancing (BiBa) strategy to process image and text features, ultimately providing an answer. Empirical results demonstrate the marked performance of ILCD on CDwPK-VQA. The primary contributions of this research are as follows:

• This research constructs a new model called ILCD for the task of crop disease VQA, addressing multiple attribute questions.
• A new dataset called CDwPK-VQA is constructed for studying crop diseases. This dataset comprises crop images, question texts, and corresponding answers.
• The informed-learning method aligns multiple modal representations of a single concept by integrating prior knowledge to guide model training, improving the performance of VQA models relying on data-driven methods.
• This research validates the proposed method through extensive experimental verification. ILCD outperforms existing multimodal methods on CDwPK-VQA. Ablation experiments demonstrating the robustness of ILCD.

This paper outlines the construction process of CDwPK-VQA in “The CDwPK-VQA dataset” section, followed by a description of ILCD in “The ILCD model” section. The Results and Discussion was showcases ILCD’s effectiveness through experiments, highlighting issues and proposing future solutions, while Conclusion provides a summary of the paper.

## Materials and Methods

### The CDwPK-VQA dataset

In this section, we outline CDwPK-VQA. We use different various visual attributes to ascertain the stage of the disease, as shown in Fig. S1. Subsequently, the answers to these questions can be used to develop appropriate treatment methods based on the onset stage. Informed learning regulates the learning behavior of the VQA model by integrating prior knowledge into the dataset, as shown in Fig. 2.

#### Data acquisition

Crop disease images are collected online using a web crawler method. These images are then curated by an agricultural expert to integrated into CDwPK-VQA. CDwPK-VQA encompasses 10 different crop varieties, namely, apple, citrus, peanut, tomato, pear, mango, papaya, olive, strawberry, and jujube, along with 19 crop diseases, namely, strawberry anthracnose, papaya anthracnose, tomato fruit cracking disease, tomato cotton blight, tomato anthracnose, citrus peel cracking disease, citrus penicillium disease, olive anthracnose, peanut leaf spot disease, pear brown rot disease, pear anthracnose disease, pear rust disease, mango anthracnose disease, apple fruit cracking disease, apple mold disease, apple water heart disease, apple anthracnose disease, jujube anthracnose disease, and jujube madness disease. Question-and-answer textual data are annotated manually with guidance from an agricultural expert, and various visual attributes are defined and depicted in Fig. 1. A dataset for crop disease triads (comprising of image, question, and answer) is constructed using the VQA dataset as a reference. Each image is manually assigned 8 visual attributes related to crop diseases and categories of questions, including condition, color, shape, texture, boundaries, number, location, size, fruit type, disease type, and leaf type. Ten candidate answers labeled with a confidence level of “yes” or “maybe” are assigned to each question, and the categories of answers are “number”, “color”, “yes/no”, and “other”. We obtained a dataset containing 708 images and 6,018 questions. Examples of CDwPK-VQA are presented in Fig. S2.

#### Data enhancement and division

Due to the limited size of the dataset, data augmentation techniques are used on images and text to expand the dataset. These techniques aim to mitigate issues related to overfitting and convergence during model training [24]. Image augmentation includes the addition of Gaussian noise, the utilization of the multiscale Retinex with chromaticity preservation (MSRCP) algorithm [25], the multiscale Retinex with color restoration (MSRCR) algorithm [26], and random image rotation. The parameters of these methods are designed as follows: (a) Gaussian noise follows a distribution with a mean of 0 and a variance of $\sigma^{2}$. The mean of 0 ensures that the brightness of the image remains unchanged and the magnitude of $\sigma^{2}$ determines the intensity of the noise, and new images are generated using $\sigma^{2}=0.1$ and $\sigma^{2}=0.01$. (b) The MSRCP and MSRCR algorithms are components of the Retinex [27] algorithm, which aims to achieve adaptive image enhancement by balancing dynamic range compression, edge enhancement, and color constancy. (c) Random image rotation is conducted within a range of 60° to 120°. The selection of parameters for these methods is determined based on empirical evidence derived from the published literature [28–30].

We enhance the question text data through 3 methods: converting all letters to lowercase, substituting and deleting keywords, and eliminating punctuation. Furthermore, we generate question samples using a sampling method [31].

Finally, the dataset follows an 8:1:1 ratio and is divided into 3 sets: the training set (1,956 images, 15,768 questions), the validation set (260 images, 2,160 questions), and the test set (260 images, 2,160 questions). All 3 datasets are annotated with the corresponding correct answers, as shown in Table 1. The question categories are classified as judgment (yes/no), counting (number), and other questions; the accuracy is assessed independently during model evaluation. It remains a challenge to achieve high performance on datasets with a limited size. To address this challenge, we integrated prior knowledge of crop diseases into the dataset.

**Table 1.** Statistical analysis of dataset division. This research utilized 3 datasets, which are VQA-CP v2, VQA-v2, and CDwPK-VQA.

Dataset name	Number of images	Number of questions
VQA-CP v2(train)	121,000	438,000
VQA-CP v2(test)	98,000	22,000
VQA-v2(train)	80,000	444,000
VQA-v2(val)	40,000	214,000
CDwPK-VQA(train)	2,228	17,948
CDwPK-VQA(test)	260	2,160
CDwPK-VQA(val)	260	2,160

#### Prior knowledge data

Prior knowledge of crop diseases is independently constructed based on the crop disease multiattribute VQA dataset. The data are presented in a ternary format (image–question–answer) following the format of the VQA dataset [21]. The dataset construction steps can be summarized as follows: (a) A curated selection of crop disease images is created, and regions of interest are annotated. (b) A crop disease object detector is trained using the fast region-based convolutional network (CNN) method on the labeled data. (c) The crop disease fast region-based CNN method identifies 272 representative images depicting various categories of crop diseases. The detected image object regions are used as inputs to the VQA model. (d) Each input image is labeled with corresponding questions and answers, ensuring alignment between the question text information and image region features. This process results prior knowledge dataset comprising 272 images and 2,180 questions. CDwPK-VQA integrates prior knowledge to expand the dataset and regulate the learning behavior of the model, as shown in Fig. 2. The dataset of CDwPK-VQA is available at https://github.com/SdustZYP/CDwPK-VQA/tree/main.

### The ILCD model

**Fig. 3.**
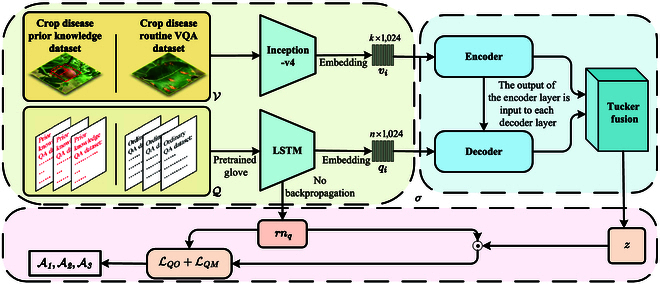
The informed-learning-guided VQA model of crop disease (ILCD) model. It leverages the introduced prior knowledge crop disease multiattribute VQA dataset (CDwPK-VQA) as its input. LSTM, long short-term memory.

This research constructs a novel ILCD. The model architecture of ILCD is shown in Fig. 3, and divided into the following steps: (a) Image features V and question features Q are extracted using a pretrained feature extraction model. (b) The coattention mechanism captures the interaction between the image and text modalities. (c) A multimodal fusion module (MUTAN) generates multimodal fusion features. (d) A BiBa strategy optimizes the features of multimodal fusion.

For a dataset D comprising n triples $\left(v_{i},q_{i},a_{i}\right),i\in \left[1,n\right]$, where $v_{i}\in V$ represents the image, $q_{i}\in Q$ represents the question, and $a_{i}\in A$ represents the answer, a function $f:V\times Q\to {\mathbb{R}}^{|A|}$ is optimized to produce accurate predictions. The image and question text encoders are $e_{v}:V\to {\mathbb{R}}^{n_{v}\times d_{v}}$ and $e_{q}:Q\to {\mathbb{R}}^{n_{q}\times d_{q}}$. Additionally, the modular coattention network (MCAN) is $\begin{array}{c} a:{\mathbb{R}}^{n_{v}\times d_{v}}\times {\mathbb{R}}^{n_{q}\times d_{q}},\;{\mathbb{R}}^{n_{q}\times d_{q}}\;\to \;{\mathbb{R}}^{n_{av}\times d_{av}},\;{\mathbb{R}}^{n_{aq}\times d_{aq}} \end{array}$, the multimodal feature fusion mechanism is $m:{\mathbb{R}}^{n_{av}\times d_{av}}\times {\mathbb{R}}^{n_{aq}\times d_{aq}}\to {\mathbb{R}}^{d_{m}}$, the deviation equilibrium strategy is $b:{\mathbb{R}}^{d_{m}}\times {\mathbb{R}}^{n_{q}\times d_{q}}\to {\mathbb{R}}^{d_{z}}$, and the classifier is $c:{\mathbb{R}}^{d_{z}}\to {\mathbb{R}}^{|A|}$. The composition of the functions is

$$f\left(v_{i},q_{i}\right),c\left(b\left(m\left(a\left(e_{v}\left(v_{i}\right),\;e_{q}\left(q_{i}\right)\right)\right),\;q_{i}\right)\right)$$ (1)

#### Question and image representations

The input to ILCD consists of global image features that are obtained from the pretrained Inception-v4 model [32]. All images are standardized to the size of 448 × 448 before extracting the features. These features are $e_{v}\left(v_{i}\right)$, where $v_{i}\in V$ represent the ith image out of n images.

The question text following a method similar to those of Kim et al. [33] and Anand et al. [34] is tokenized into words. Every question text retains a maximum of 12 words. Utilizing 300-D Glove word embeddings [35] transforms each word in the question into a word vector. This results in a sequence of words with a size of m×300, where m∈[1,12] is the question length. A long short-term memory (LSTM) network [36] with a single layer $e_{q}$ processes the word embeddings generated by Glove for the ith question. This processing produces a feature matrix $e_{q}\left(q_{i}\right)$, where $q_{i}\in Q$.

We use zero padding [37] to address the issue of variable question text lengths m. The vector is extended to the maximum size (m=14). During the training process, we utilize logarithm padding along with a −∞ to each softmax layer. This method effectively mitigates the occurrence of underflow questions.

#### Attention mechanism

MCAN consists of a stack of L layers of modular coattention (MCA) layers. Each MCA layer integrates 2 fundamental attention units: the self-attention (SA) unit and the guided-attention (GA) unit, utilizing a modular combination of an encoder–decoder structure. The design of these fundamental attention units is influenced by the scaled dot–product attention mechanism introduced by Vaswani et al. [38].

##### SA and GA units

The inputs q:query, k:key, and v:value are derived from distinct mappings of the input vectors in the scaled dot–product attention mechanism. For each vector $q\in {\mathbb{R}}^{1\times d_{k}}$ to compute the score, where this is the fraction used to calculate self-attention s=q⋅k. One instance of $q\in {\mathbb{R}}^{1\times d_{k}}$ corresponds to n key–value pairs n×(k,v), where $k\in {\mathbb{R}}^{1\times d_{k}},v\in {\mathbb{R}}^{1\times d_{k}}$, encapsulated into a key matrix $K\in {\mathbb{R}}^{n\times d_{k}}$ and a value matrix $V\in {\mathbb{R}}^{n\times d_{k}}$. We normalize s by dividing by $\sqrt{d_{k}}$ to ensure stable gradients. Subsequently, the softmax activation function is applied to derive the attention weights of s. Finally, we obtain the output of scaled dot–product attention by multiply attention weights with v and summing the results:

$$A\left(Q,K,V\right)=\text{softmax}\left(\frac{QK}{\sqrt{d_{k}}}\right)V$$ (2)

We concatenate multiple instances of scaled dot–product attention to enhance the characterization of the attended feature, resulting in the output feature f:

$$f=MA\left(Q,K,V\right)=\text{Concat}\left[h_{1},h_{2},\cdots h_{h}\right]W^{o}$$ (3)

$$h_{i}=A\left(QW_{i}^{Q},\;KW_{i}^{K},VW_{i}^{V}\right)$$ (4)

Here, $W_{i}^{Q},W_{i}^{K},W_{i}^{V},W^{o}$ is the projection matrix.

**Fig. 4.**
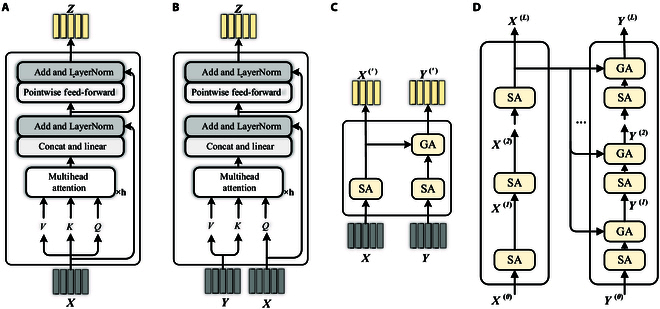
Components of the attention mechanism. (A) Self-attention (SA) unit. (B) Guided-attention (GA) unit. (C) Modular coattention (MCA) layer. (D) Modular coattention network (MCAN).

Implementing SA and GA requires the utilization of a multihead attention mechanism. Each SA and GA unit consists of 2 sublayers, namely, a multihead sparse attention layer and a pointwise fully connected feed-forward layer. We denote a set of visual features $e_{v}\left(v_{i}\right)$ as $X=\left[x_{1},\ldots ,x_{n}\right]$ and a set of question text features $e_{q}\left(q_{i}\right)$ as $Y=\left[y_{1},\ldots ,y_{m}\right]$. The SA unit takes X as input, as shown in Fig. 4A. Similarly, the GA unit takes X and Y as input, as shown in Fig. 4B. Y guides the attentional learning of X to model the pairwise relationship between X and Y.

##### MCA layer

We construct the MCA layer by utilizing the fundamental attention units, as shown in Fig. 4C. Stacking these MCA layers, the output of one MCA layer becomes the input of the next MCA layer, resulting in a deep cascade of these layers where the number of input features matches the number of output features.

The MCA layer is designed to capture the interactions between word pairs $y_{i},y_{j}$ within the question text, as well as the interactions between image region pairs $x_{i},x_{j}$. Additionally, it captures the interactions across multiple modalities of $x_{i},y_{j}$, where $x_{i}\in X,\;y_{j}\in Y$, as shown in Fig. 4C.

##### Deep coattention network

The visual feature X and the question text feature Y are input into MCAN through L stacked layers ($MCA^{\left(1\right)},\ldots ,MCA^{\left(L\right)}$), as shown in Fig. 4D. The structure of MCAN is similar to that of an encoder–decoder architecture. The details of the encoder and decoder are outlined in the following:

• The encoder consists of N layers of SA units for learning detailed image features. Initially, the SA unit takes visual features $X=\left[x_{1},\ldots ,x_{n}\right]$ as input. The multihead sparse attention layer captures correlations between each $\langle x_{i},x_{j}\rangle i,j\in \left[1,\;n\right]$. Then, a fully connected feed-forward layer is applied to each point in order to further refine the output of the image region of interest. Each SA unit receives input from the output of the preceding SA unit. The output of the encoder is $X\left(N\right)=\left[x_{1}^{N},\ldots ,x_{n}^{N}\right]$, as shown on the left-hand side of Fig. 4D.
• The decoder consists of N sets of stacked SA and GA units. It operates in a similar manner to the encoder but with a focus on question-guided learning, when provided with a set of question text features $Y=\left[y_{1},\ldots ,y_{m}\right]$ as its input. The output of the encoder $X\left(N\right)=\left[x_{1}^{N},\ldots ,x_{n}^{N}\right]$ is fed into the GA unit along with the output of the preceding SA unit. A linear transformation aligns the visual feature dimension with the question text feature dimension. The input to each SA and GA unit includes the output of the preceding SA and GA unit and the encoder. This method allows question-oriented learning of the visual features of the input and learning the relevance between each $\langle x_{i},y_{j}\rangle i\in \left[1,n\right],j\in \left[1,m\right]$. The output of the decoder is $Y\left(N\right)=\left[Y_{1}^{N},\ldots ,Y_{m}^{N}\right]$, as shown on the right-hand side of Fig. 4D.

The input features of ${\mathrm{MCA}}^{\left(l\right)}$ are $X^{\left(l-1\right)}$ and $Y^{\left(l-1\right)}$. The output features are $X^{\left(l\right)}$ and $Y^{\left(l\right)}$. These features are fed into ${\mathrm{MCA}}^{\left(l+1\right)}$. The feature transfer relationship across different MCA layers is as follows:

$$\left[X^{\left(l\right)},Y^{\left(l\right)}\right]={\mathrm{MCA}}^{\left(l\right)}\left(\left[X^{\left(l-1\right)},Y^{\left(l-1\right)}\right]\right)$$ (5)

#### Multimodal feature fusion

Bilinear models are becoming increasingly popular as solutions for the multimodal feature fusion in VQA tasks. However, the number of parameters in these models frequently impacts the performance of graphics processing units. Consequently, we use Tucker decomposition to manage the fused tensor T. The resulting core tensor $T_{c}$ from the decomposition is used in the training of the model. This method reduces the number of parameters and preserving the essential features.

##### Tucker decomposition

Given 3-way tensor $T\in {\mathbb{R}}^{d_{q}\times d_{v}\times |A|}$, we use the Tucker decomposition to express T in terms of factor matrices $W_{q}$, $W_{v}$, and $W_{o}$, along with a core tensor $T_{c}$:

$$T=\left(\left(T_{c}\times W_{q}\right)\times W_{v}\right)\times W_{o}$$ (6)

Here, $W_{q}\in {\mathbb{R}}^{d_{q}\times t_{q}}$, $W_{v}\in {\mathbb{R}}^{d_{v}\times t_{v}}$, and $W_{o}\in {\mathbb{R}}^{d_{o}\times t_{o}}$, while $T_{c}\in {\mathbb{R}}^{t_{q}\times t_{v}\times t_{o}}$. We customarily represent T as $T\;=\;\left[T_{c},W_{q},W_{v},W_{o}\right]$. For a comprehensive discussion of Tucker decomposition and tensor analysis, refer to Kolda and Bader [39].

##### Multimodal Tucker fusion

We parameterize the weights of tensor T by utilizing the Tucker decomposition, and the tensor is

$$T=\left(\left(T_{c}\times q^{⊤}W_{q}\right)\times v^{⊤}W_{v}\right)\times W_{o}$$ (7)

Here, $q^{⊤}W_{q}$ is the projection of visual features. $v^{⊤}W_{v}$ is the projection of question text features. The full bilinear interaction between 2 modal features is z utilized for predicting the answer. Given the definitions $\tilde{v}=v^{⊤}W_{v}$, $\tilde{v}\in {\mathbb{R}}^{t_{v}}$, and $\tilde{q}=q^{⊤}W_{q}$, $\tilde{q}\in {\mathbb{R}}^{t_{q}}$, to obtain the multimodal features of fusion:

$$z=\left(T_{c}\times \tilde{q}\right)\times \tilde{v},z\in {\mathbb{R}}^{t_{o}}$$ (8)

Subsequently, the resulting z is projected into the prediction space $y=z^{⊤}W_{o}$, where $y\in {\mathbb{R}}^{|A|}$, and the probability distribution of the outcome is computed using $p=\text{softmax}\left(y\right)$. The experiments utilize nonlinear activation functions $\tilde{v}=\text{tanh}\left(v^{⊤}W_{v}\right)$, where $\tilde{v}\in {\mathbb{R}}^{t_{v}}$ and $\tilde{q}=\text{tanh}\left(q^{⊤}W_{q}\right)$, $\tilde{q}\in {\mathbb{R}}^{t_{q}}$, which demonstrate improved results.

#### BiBa learning strategy

**Fig. 5.**
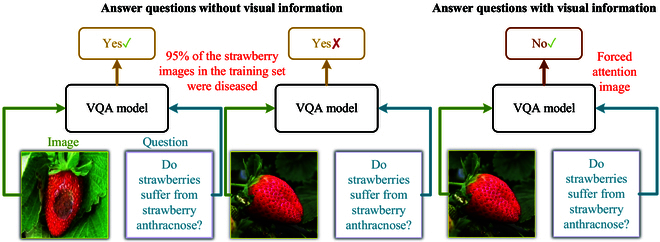
Bias-balancing (BiBa) strategy. In order to reduce the tendency of the VQA model to rely on statistical patterns in generating answers, redirect the focus of attention to image information during the training process. This method will assist in reducing the inherent unimodal bias present in the dataset.

Despite achieving superior performance compared to human inference on specific benchmarks [40] after being trained on extensive datasets, VQA models often fail to adequately consider image information during training. Instead, models rely on statistical patterns that exist between question structures and answer frequencies for inference [41,42]. The majority of strawberry disease images in the dataset depict strawberry anthracnose. However, the VQA model does not thoroughly analyze image details to accurately determine whether strawberries are diseased. Consequently, the model may predict that “the fruit is diseased” when queried about the health of strawberries despite the existence of healthy strawberry images. Addressing this statistical unimodal bias in dataset construction is a challenging task, as shown in Fig. 5. Therefore, we propose a BiBa strategy (BiBa). BiBa aims to oblige the VQA model to prioritize learning visual features from crop disease images and reduce the influence of answer statistical patterns.

BiBa aims to establish a specialized branch for question text analysis. This branch captures variations in question text representations in order to impact the predictions of the main model, represented as $f_{Q}:Q\to {\mathbb{R}}^{|A|}$; the dedicated question text branch extracts the ith text feature $e_{q}\left(q_{i}\right)$, labeled as $n_{q}$, through a text encoder $e_{q}$. This branch includes a matrix mapping neural network $rn_{q}:{\mathbb{R}}^{n_{q}\times d_{q}}\to {\mathbb{R}}^{|A|}$ and a classifier $c_{q}:{\mathbb{R}}^{|A|}\to {\mathbb{R}}^{|A|}$, denoted as

$$f_{Q}\left(q_{i}\right)=c_{q}\left(rn_{q}\left(e_{q}\left(q_{i}\right)\right)\right)$$ (9)

The parameters of the dedicated question text branch are removed after the completion of model training. The question text features from the branches are processed by the matrix mapping neural network $rn_{q}:{\mathbb{R}}^{n_{q}\times d_{q}}\to {\mathbb{R}}^{|A|}$. The outputs of $rn_{q}$ through sigmoid function processing to yield $m\in \left(0,1\right)$, which is to convert the original values between [0,1] using a nonlinear activation function, with m squared to obtain the mask $m^{2}$. Subsequently, the loss is adjusted dynamically by appropriately modifying the prediction of the VQA model through the $z⊙m^{2}$ operation. The overall model is $f_{QM}$:

$$f_{\mathrm{QM}}=z⊙{\left(\sigma \left({\mathrm{rn}}_{q}\left(e_{q}\left(q_{i}\right)\right)\right)\right)}^{2}$$ (10)

Here, the function σ(⋅) refers to the sigmoid function. σ(⋅) is utilized to map variables within the range of $\left[0,1\right]$. We use σ(⋅) to generate the masks.

#### Joint learning procedure

Two loss-computing gradients are utilized to co-optimize the parameters of the model and dedicated question text branches to obtain z. The primary loss $L_{\mathrm{QM}}$ corresponds to the binary cross-entropy loss associated with the prediction of $f_{QM}$, where $\theta_{\mathrm{QM}}$ represents all parameters that contribute to $L_{QM}$. The loss of the question text branch is $L_{QO}$. $L_{QO}$ is the cross-entropy loss used to predict $f_{Q}\left(q_{i}\right)$. The parameters $\theta_{\mathrm{QO}}$ are the dedicated question text branch. The question text features $e_{q}\left(q_{i}\right)$ input into the dedicated question text branch are identical to those input into the main model. The loss of the dedicated question text branch is excluded from backpropagating to $e_{q}$ in order to prevent $e_{q}$ from directly learning the unimodal bias. The final model loss is obtained by joining losses $L_{\text{ILCD}}$:

$$L_{\text{ILCD}}\left(\theta_{QM},\theta_{QO},D\right)=L_{QM}\left(\theta_{QM},D\right)+L_{QO}\left(\theta_{QO},D\right)$$ (11)

## Results and Discussion

### Datasets

• *VQA-v2.* This is a widely used VQA benchmark dataset [43]. Each image is associated with 3 questions. Each question is associated with 10 answers. The VQA-v2 dataset consists of 3 subsets: train (80,000 images and 444,000 question–answer [QA] pairs), val (40,000 images and 214,000 QA pairs), and test (80,000 images and 448,000 QA pairs), as shown in Table 1. The distribution of answers between the train and val datasets of VQA-v2 varies markedly. This indicates a marked unimodal bias in the dataset.
• *VQA-CP v2*. Based on VQA-v2 constructs, VQA-CP v2 [41] is used to evaluate the robustness of the VQA model. The main objective of VQA-CP v2 is to address the discrepancy in answer distribution by reducing unimodal bias. VQA-CP v2 consists of 2 subsets: train (121,000 images and 438,000 QA pairs) and test (98,000 images and 22,000 QA pairs). The detailed information of VQA-CP v2 is shown in Table 1.
• *CDwPK-VQA.* CDwPK-VQA is a multiattribute VQA dataset specifically designed to incorporate prior knowledge related to crop diseases. It includes 10 different crops, including apple, citrus, peanut, tomato, and date. Additionally, it encompasses 19 crop diseases, such as apple anthracnose, citrus penicillium, peanut leaf spot, tomato mildew, and date anthracnose. CDwPK-VQA consists of 3 segments: train (2,200 images and 18,000 QA pairs), val (260 images and 2,160 QA pairs), and test (260 images and 2,160 QA pairs). The detailed information of CDwPK-VQA is shown in Table 1.

Performance comparison experiments are conducted in this research using the VQA-CP v2, VQA-v2, and CDwPK-VQA datasets. All ablation experiments are performed on CDwPK-VQA.

### Implementation details

The experimental parameters of the model are detailed in Table S1. The experiments are conducted on the Ubuntu 20.04 platform using Python 3.8. The model implementation utilizes PyTorch-1.11.0 with cuda version 11.3. The experiments are performed on an NVIDIA 3090 graphics card with 24 GB of onboard memory. The model training time spent is 6.2 h.

### Evaluation metrics

The questions in our dataset are classified into 3 categories: yes/no, number, and other. Accuracies are calculated for each category and the entire dataset. The true positives are TP, the true negatives are TN, and N represents the total number of samples (N=TP+TN+FP+FN). False positives are FP, and false negatives are FN. Accuracy is defined as the ratio of correctly classified samples to the total number of samples. Accuracy can be calculated using the following formula:

$$\text{Accuracy}=\frac{\left(TP+TN\right)}{N}$$ (12)

### Performance comparisons

In Table 2, we evaluate the performance of ILCD through comparative experiments on VQA-v2, VQA-CP v2, and CDwPK-VQA. Existing models are categorized into 5 main categories based on the methods of innovation: I, typical VQA models (such as stacked attention network [33], hierarchical attention network [44], generalized VQA [41], and language and cross-modal encoding representations from transformers [45]); II, reweighting-based models (such as suppressing biased samples [46] and language-refined salience [47]); III, integration-based models (such as recurrent underlying bipartite model [48], graphical regularization learning [49], deep learning projection [50], late fusion with product of experts [51], and graphical Gaussian embedding [52]); IV, models based on manual annotation (such as hierarchical interactive network for text and image [53] and semantic concept recognition [54]); V, models based on counterfactuals and comparative learning (such as cross-modal semantic similarity [55], commonsense-based VQA [56], explicit content and distribution [57], audio-visual modality [58], and cross-modal knowledge contrastive learning [59]).

**Table 2.** Performance comparisons. This table compares the performance of the models for different innovation directions using 2 public datasets and CDwPK-VQA. The performance of the different models on the dataset is categorized into “all”, “yes/no”, “number”, and “other” to calculate the accuracy rate. The optimal values obtained by ILCD are represented by bolded numbers. The suboptimal values are represented by underlined numbers.

Categories	Model	VQA-CP v2(test) (%)				VQA-v2(val) (%)				CDwPK-VQA(test) (%)			
		All	Yes/no	Number	Other	All	Yes/no	Number	Other	All	Yes/no	Number	Other
I	SAN [33]	24.96	38.35	11.14	21.74	52.41	70.06	39.28	47.84	66.58	79.82	66.37	75.61
	HAN [44]	28.65	52.25	13.79	20.33	ND	ND	ND	ND	68.29	80.19	68.52	74.59
	GVQA [41]	31.30	57.99	13.68	22.14	48.24	72.03	31.17	34.65	71.93	81.32	67.95	76.82
	LXMERT [45]	46.23	42.84	18.19	55.51	ND	ND	ND	ND	81.46	90.52	71.86	81.47
II	SBS [46]	42.21	53.51	11.12	44.82	63.84	81.81	43.03	55.67	77.54	83.17	74.68	81.31
	LRS [47]	49.45	72.36	10.93	48.02	62.20	78.80	41.60	54.40	83.68	93.09	70.35	82.45
III	RUBi [48]	45.42	63.03	11.91	44.33	58.19	63.04	41.00	54.43	79.26	86.71	75.25	76.34
	GRL [49]	42.33	59.74	14.78	40.76	ND	ND	ND	ND	78.75	85.67	70.63	81.42
	DLP [50]	48.87	70.99	18.72	45.57	57.96	76.82	39.33	48.54	83.17	95.16	71.38	82.85
	LPF [51]	55.34	88.61	23.78	46.57	55.01	64.87	37.45	52.08	84.93	95.08	73.50	82.27
	GGE [52]	57.32	87.04	27.75	49.59	59.11	73.27	39.99	54.39	83.64	94.62	72.39	83.14
IV	HINT [53]	46.73	67.27	10.61	45.88	63.38	91.18	42.99	55.56	80.14	94.81	70.68	81.10
	SCR [54]	49.45	72.36	10.93	48.02	ND	ND	ND	ND	84.80	95.92	74.01	82.44
V	CSS [55]	41.16	43.96	12.78	47.48	ND	ND	ND	ND	78.14	80.33	72.51	83.68
	CF-VQA [56]	49.74	74.81	18.46	45.19	63.73	82.15	44.29	54.86	79.77	91.80	77.43	79.84
	ECD [57]	41.78	42.74	14.89	48.66	ND	ND	ND	ND	78.79	82.77	73.59	84.41
	AVM [58]	40.08	42.51	12.36	46.41	63.87	81.43	43.82	55.82	80.45	90.82	76.89	81.86
	CKCL [59]	**55.05**	**90.33**	18.99	46.46	62.55	79.17	41.94	55.38	79.94	88.41	74.69	81.45
Ours	ILCD	49.75	68.89	**21.26**	**48.83**	**68.90**	**87.94**	**46.58**	**59.03**	**86.06**	**96.94**	**79.80**	**85.65**

ND, no data; SAN, stacked attention network; HAN, hierarchical attention network; GVQA, generalized VQA; LXMERT, language and cross-modal encoding representations from transformers; SBS, suppressing biased samples; LRS, language-refined salience; RUBi, recurrent underlying bipartite model; GRL, graphical regularization learning; DLP, deep learning projection; LPF, late fusion with product of experts; GGE, graphical Gaussian embedding; HINT, hierarchical interactive network for text and image; SCR, semantic concept recognition; CSS, cross-modal semantic similarity; CF-VQA, commonsense-based VQA; ECD, explicit content and distribution; AVM, audio-visual modality; CKCL, cross-modal knowledge contrastive learning

**Fig. 6.**
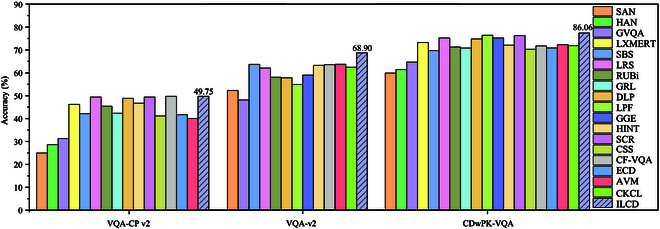
Visualization of comparative experiments. The performance of the participating models on “VQA-CP v2”, “VQA-v2”, and “CDwPK-VQA” is visualized from left to right.

The results of our comparative experiments demonstrate that ILCD outperforms the majority of existing VQA models on 3 datasets. As shown in Table 2, ILCD achieves the highest performance on the VQA-CP v2 dataset for the categories of “number” and “other”. These 2 categories of questions reflect the ability of the VQA model to address unimodal bias. However, ILCD did not achieve state-of-the-art performance on the “yes/no” category of questions. The reason can be attributed to answering “yes/no” questions requiring the VQA model to learn semantic information about the content inside and outside of the image. Additionally, correctly answering the VQA-CP v2 dataset necessitates a substantial amount of external knowledge. However, ILCD lacks access to the external knowledge. As a result, the model encounters difficulties in accurately answering such questions. On the VQA-v2 dataset, ILCD achieves the highest performance in the categories of “number”, “other”, and “yes/no” when confronted with open-domain questions. The results demonstrate the effectiveness and robustness of the model. Additionally, the accuracy of the data regarding various visual attributes in CDwPK-VQA is calculated separately, as shown in Table 3. ILCD demonstrates the highest accuracy rate in the yes/no” category. This outcome substantiates the model’s profound semantic comprehension of crop disease images, as well as the optimal ability of ILCD to address questions related to “number” and “other” categories. The results demonstrate the practical applicability of ILCD. Statistically, ILCD exhibited high performance on all 3 datasets. We visualized the comparison experiments, as shown in Fig. 6, and selected 5 models for comparison of heat map visualizations, as shown in Fig. S3.

**Table 3.** Performance comparison of ILCD under various visual attribute questions on CDwPK-VQA. The use of bold indicates optimal performance, and the use of underlining indicates secondary performance.

Visual attributes	Accuracy (%)	Visual attributes	Accuracy (%)
Condition	86.12	Location	85.25
Color	87.06	Size	87.82
Shape	87.23	Fruit type	85.07
Texture	84.66	Disease type	86.50
Boundaries	85.29	Leaf type	87.39
Number	78.80	Other	**90.52**

### Ablation studies

We conduct a series of comprehensive ablation experiments to investigate the impact of different modules in ILCD on performance. In this paper, ablation experiments are categorized into 3 categories: (a) the impact of prior knowledge on model performance, (b) the experimental analysis of the coattention mechanism, MUTAN, and BiBa, and (c) the evaluation of the impact of different image and text feature extractors on model performance.

We design the following experiments based on the logical relationship between the modules and the importance to ILCD: “the influence of prior knowledge”, “different coattention mechanism structures”, “diverse MCAN layers”, “different multimodal fusion mechanisms”, “different strategies for unimodal bias”, “different image feature extractors”, and “impact of different text encoders”.

#### The influence of prior knowledge

**Fig. 7.**
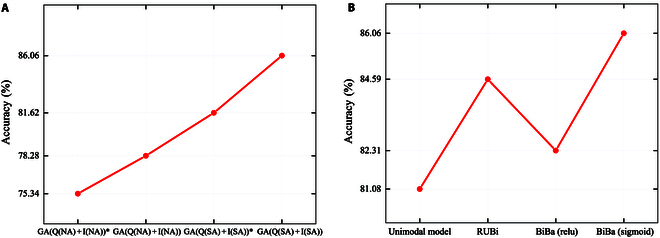
Visualization of the results from ablation experiments. The visualization in (A) and (B) depicts ablation experiments conducted on introduction of prior knowledge and different strategies for unimodal bias.

The process of establishing prior knowledge emulates the image reasoning of the ILCD based on a question. MCAN captures the intricate interaction between the 2 modalities. The prior knowledge enhances the performance of MCAN by aligning diverse modal representations of the same concept. To validate this, we conduct experiments on 2 MCA layers (GA(Q(NA) + I(NA))), (GA(Q(SA) + I(SA))), as shown in Table 4 and Fig. 7A. “${}^{*}$” denotes the absence of prior knowledge. The results demonstrate that the performance of the model is further enhanced by the introduction of prior knowledge through ILCD, particularly when SA is utilized and a performance is achieved. This confirms the effectiveness of a prior knowledge.

**Table 4.** The influence of prior knowledge on model performance. “${}^{*}$” indicates that no prior knowledge is involved in the training. The use of bold indicates optimal performance, and the use of underlining indicates secondary performance.

Model	All	Yes/no	Number	Other
GA(Q(NA) + I(NA))*	75.34	94.56	73.86	74.91
GA(Q(NA) + I(NA))	78.28	96.85	75.76	75.65
GA(Q(SA) + I(SA))*	81.62	95.79	78.31	81.90
GA(Q(SA) + I(SA))	**86.06**	**96.94**	**79.80**	**85.65**

#### Different coattention mechanism structures

The experiments in Table 5 assess the effects of different coattention mechanism structures on model performance. In this table, “Q” represents text features and the “I” represents image features. “NA” denotes modal encoding without SA, and “SA” denotes modal encoding with SA. “GA” represents GA and represents comprehensive interaction modeling of multimodalities.

**Table 5.** The performance of using different coattention mechanism structures. The use of bold indicates optimal performance, and the use of underlining indicates secondary performance.

Model	All	Yes/no	Number	Other
Q(NA) + I(NA)	76.82	95.28	76.85	73.78
Q(SA) + I(NA)	78.03	95.91	76.60	76.04
GA(Q(NA) + I(NA))	78.28	96.85	75.76	75.65
GA(Q(SA) + I(NA))	81.37	96.77	77.84	80.83
GA(Q(NA) + I(SA))	84.09	96.81	78.49	84.27
GA(Q(SA) + I(SA))	**86.06**	**96.94**	**79.80**	**85.65**

#### Diverse MCAN layers

**Fig. 8.**
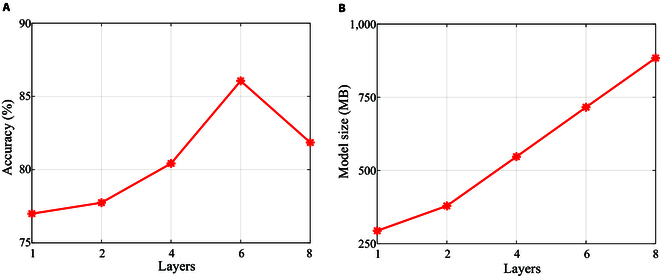
Line plots of modular coattention network (MCAN) ablation experiments with different numbers of layers. The analysis focuses on the relationship between the number of layers in MCAN and the trend of model accuracy (A) and the number of parameters (B).

**Fig. 9.**
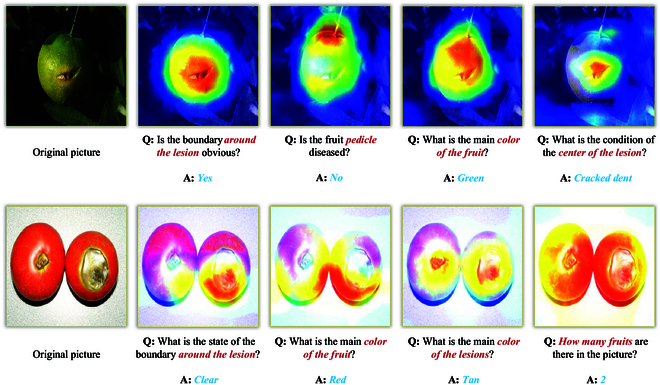
Model attention visualization. The model adjusts the region of attention in the image based on different questions.

The impact of different numbers of MCAN layers on model performance is investigated. Figure 8A displays the accuracy of the model for different values of L (number of MCAN layers) alongside the corresponding model parameters. Figure 8B demonstrates that the performance of the model improves as the number of MCAN layers increases. The accuracy reaches its best performance when L=6 before declining with additional layer increments. The important influence of MCANs with different layer counts on model performance highlights the crucial role in the model. Considering accuracy and resource utilization, we determine L=6 as the optimal number of attention layers for this research. Figure 9 presents the attention visualization of the model with the optimal number of MCAN layers.

#### Different multimodal fusion mechanisms

The experiment investigates the influence of multimodal feature fusion methods on model performance by comparing the MUTAN method with 3 other fusion methods: (a) concatenation, which refers to a simple addition of multimodal features; (b) multiplication, which entails a basic multiplication of multimodal features; and (c) multimodal low-rank bilinear pooling, which refers to element-by-element multiplication after projecting different modal features into a low-dimensional output space. As shown in Table 6, the performance of MUTAN is compared using different activation functions, namely, tanh and relu. Experimental results demonstrate that MUTAN outperforms other bilinear fusion methods. Full bilinear interactions are better in MUTAN between low-dimensional projections compared to the simplistic feature fusion of other methods. Additionally, the utilization of the tanh activation function greatly improves the performance of the model. This highlights the capability of MUTAN to effectively capture multimodal fusion features using tanh.

**Table 6.** The performance of using different multimodal fusion mechanisms. The use of bold indicates optimal performance, and the use of underlining indicates secondary performance.

Model	Activation	All	Yes/no	Number	Other
Multiplication	tanh	73.70	89.44	68.91	71.93
Concatenation	tanh	75.17	88.65	68.15	74.15
MLB	tanh	77.48	95.28	78.37	74.39
MUTAN	relu	80.21	96.07	75.22	78.44
MUTAN	tanh	**86.06**	**96.84**	**79.80**	**85.65**

#### Different strategies for unimodal bias

The experiments presented in Table 7 and Fig. 6 delineate the impact of various strategies for managing unimodal biases on model performance. We emphasize the importance of addressing unimodal biases by comparing the BiBa strategy with other regular models (lacking a specific strategy for unimodal biases). We establish the effectiveness of our proposed strategy by comparing it with the recurrent underlying bipartite model strategy. The experimental results of the BiBa strategy utilizing different activation functions confirm the effectiveness of sigmoid-generated masks. Table 7 provides evidence of the exceptional capability of the BiBa strategy in mitigating unimodal bias. BiBa’s method of mitigating the impact of dataset distribution through masking is effective.

**Table 7.** The performance of using different strategies for unimodal bias. The use of bold indicates optimal performance, and the use of underlining indicates secondary performance.

Model	Activate	All	Yes/no	Number	Other
Unimodal model	None	81.08	95.36	78.14	79.42
RUBi	sigmoid	84.59	96.24	78.73	82.01
BiBa	relu	82.31	95.65	77.94	84.16
BiBa	sigmoid	**86.06**	**96.94**	**79.80**	**85.65**

#### Different image feature extractors

To ensure the validity of the visual features input to the model, we compare 6 image feature extractors: fbresnet, hsresnet, Inception-v2, Vgg19, Resnet152, and Inception-v4. The results presented in Table S2 demonstrate that the Inception-v4 model surpasses the other models in terms of performance. The results indicate that the Inception-v4 model exhibits superior effectiveness in extracting visual features. Additionally, we evaluate the jointed influence of prior knowledge and feature extractors. Therefore, we compare the performance of the Inception-v4 model with and without prior knowledge during training (indicated by “*”). The results in Table S2 highlight the important performance enhancement achieved with prior knowledge, demonstrating that the utilization of a rational image feature extractor and prior knowledge enhances the model’s comprehension of images.

#### Impact of different text encoders

We conduct ablation experiments to evaluate the efficacy of LSTM with large language models. The experiments are designed to compare the performance and number of parameters of LSTM, the T5 model, and a traditional CNN model. The results presented in Table S3 demonstrate that the performance of the LSTM model is comparable to that of the T5 model for text lengths exceeding 12. However, the T5 model has a markedly higher number of parameters compared to the LSTM model. Therefore, consider that the performance of the model and the arithmetic overhead are important when evaluating the suitability of the LSTM model for the crop disease VQA task. LSTM is deemed suitable based on the experimental results.

### Qualitative analysis

**Fig. 10.**
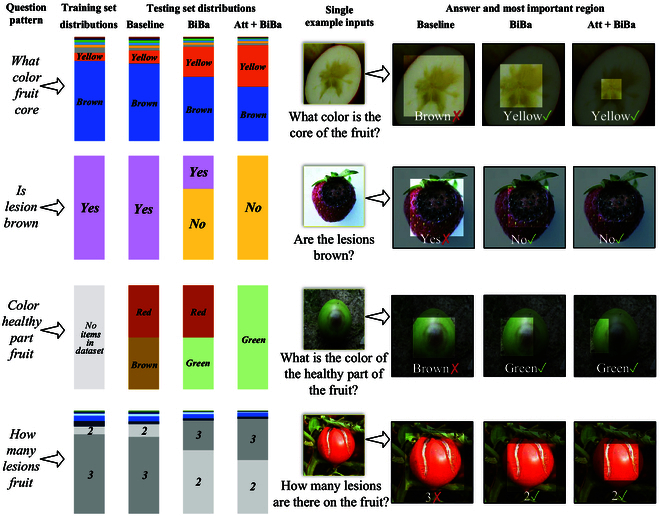
Qualitative analysis of model performance. The left side presents the answer distributions for the CDwPK-VQA training set, the baseline model evaluated on the test set, and the answer distributions for ILCD. On the right side, the attention heat map for different models is displayed across various question patterns. Att, attention.

To enhance our comprehension of the influence of the attention mechanism and unimodal BiBa strategy on the models, we analyze the distributions of answers for specific question patterns of ILCD in CDwPK-VQA, as shown in Fig. 10. This comparison includes the baseline VQA model (without the attention mechanism and unimodal BiBa strategy), the model with the BiBa strategy, and the model with the coattention mechanism and the BiBa strategy. The examples used for illustration are extracted from [60]. (a) In the first row of Fig. 10, the question pattern relates to the color of apple hydatid disease. Despite 90% of such questions in the training set being answered as brown, the color of the image in the test set is yellow. BiBa enables the VQA model to take into account the realistic visual features, which are further enhanced by the attention mechanism. In contrast, the baseline VQA model disregards the realistic visual features. (b) In the second row, the judgment question regarding the color of strawberry anthracnose tends to output “yes”, but the BiBa strategy and the attention mechanism align with the realistic visual features, where the correct answer is “no”. This discrepancy is attributed to the presence of unimodal bias. (c) In the third row, ILCD effectively manages unseen questions, demonstrating its ability to build and generalize visual and textual concepts from other models. (d) In the fourth row, ILCD shows an understanding of the relationship between spot area and question text, whereas the focus of the baseline VQA model remains on the fruit.

### Discussion

The above experiments demonstrate that the proposed ILCD surpasses other existing multimodal models. As shown in Fig. S4, ILCD obtains visual attribute descriptions of disease images from various questions in the dataset. These descriptions are then utilized to identify the stage of disease onset and provide specific control strategies. Figures 9 and 10 also demonstrate the exceptional performance of ILCD. ILCD utilizes Inception-v4 and LSTM as image and text encoders to enhance the capability of feature extraction. These encoders extract the complete feature information from the crop disease images and question texts present in the dataset. Additionally, the coattention mechanism effectively manages the interactions between images and text, associates the keywords of the question text with the regions of crop disease images, thereby efficiently extracting fine-grained features. Moreover, the multimodal feature fusion model (MUTAN) used in ILCD has the capability to extract essential core features from the fused multimodal features. MUTAN reduces the computational complexity of the model while preserving the essential information. Lastly, ILCD adjusts the learning behavior of the model using the BiBa strategy to focus on the image features of crop diseases. BiBa reduces the dependence on statistical rules of related questions in the dataset, improving the reliability and universality of ILCD. The ability of ILCD to address various inquiries regarding visual attributes in Fig. S4 stems from our constructed dataset CDwPK-VQA. CDwPK-VQA includes 19 common crop disease categories and textual annotations of various visual attributes. ILCD provides solutions for diagnosing and managing diseases at different stages.

Nonetheless, ILCD exhibits certain limitations in its ability to effectively manage previously unseen crop disease images. The model is incapable of understanding or recognizing images of disease species that are not included in the dataset. To address this limitation, future research will focus on developing a zero-shot VQA model.

This method aims to provide accurate answers to questions that are not present in the dataset. Ultimately, the zero-shot method improves the ability of the VQA model to generalize.

## Conclusion

In this paper, we construct a novel VQA model called ILCD. ILCD is designed to extract descriptions of various attributes (such as color, shape, size, and state) from images of crop diseases in order to determine the stage at which the disease begins. We validate the superior performance of ILCD through a series of extensive experiments. ILCD consists of 5 modules: an image feature extractor (Inception-v4), a question text feature extractor (LSTM), coattention, multimodal feature fusion (MUTAN), and a BiBa strategy. The primary contribution of this research is the detailed modeling of both modalities through the utilization of the coattention mechanism within the encoder–decoder structure. Subsequently, MUTAN fuses the coattended features to reduce the feature size. Lastly, BiBa is used to enhance image-based learning to reduce unimodal bias in the VQA model. Additionally, we incorporate an informed-learning method to integrate prior knowledge into CDwPK-VQA. The prior knowledge enables ILCD to achieve optimal performance on CDwPK-VQA. Comparative experiments demonstrate that ILCD outperforms existing VQA models overall. Ablation experiments demonstrate the following findings: (a) The informed-learning method enhances the performance of ILCD by 4.44%. (b) Inception-v4 and LSTM achieve superior results while maintaining a compact model size. (c) The optimal number of coattention layers is 6. (d) MUTAN shows a performance improvement of 12.36% compared to the simple multiplication fusion method. (e) The BiBa strategy enhances model performance by 4.98%. This research presents innovative solutions for the identification of crop diseases and the diagnosis of disease stages. ILCD contributes to the advancement of intelligent agriculture by reducing the need for manual labor and effectively managing diseases in agricultural production.

## Data Availability

The datasets generated during and/or analyzed during the current research are available from the corresponding authors on reasonable request.
