# Supplementary material for: Informed-Learning-Guided Visual Question Answering Model of Crop Disease
Source: Plant Phenomics. 2024 Dec 16;6:0277. doi: 10.34133/plantphenomics.0277 (PMC11649200; doi:10.34133/plantphenomics.0277)
Supplement: Supplementary 1 — Figs. S1 to S4 Tables S1 to S3 [file plantphenomics.0277.f1.zip › Fig-S1.pdf]

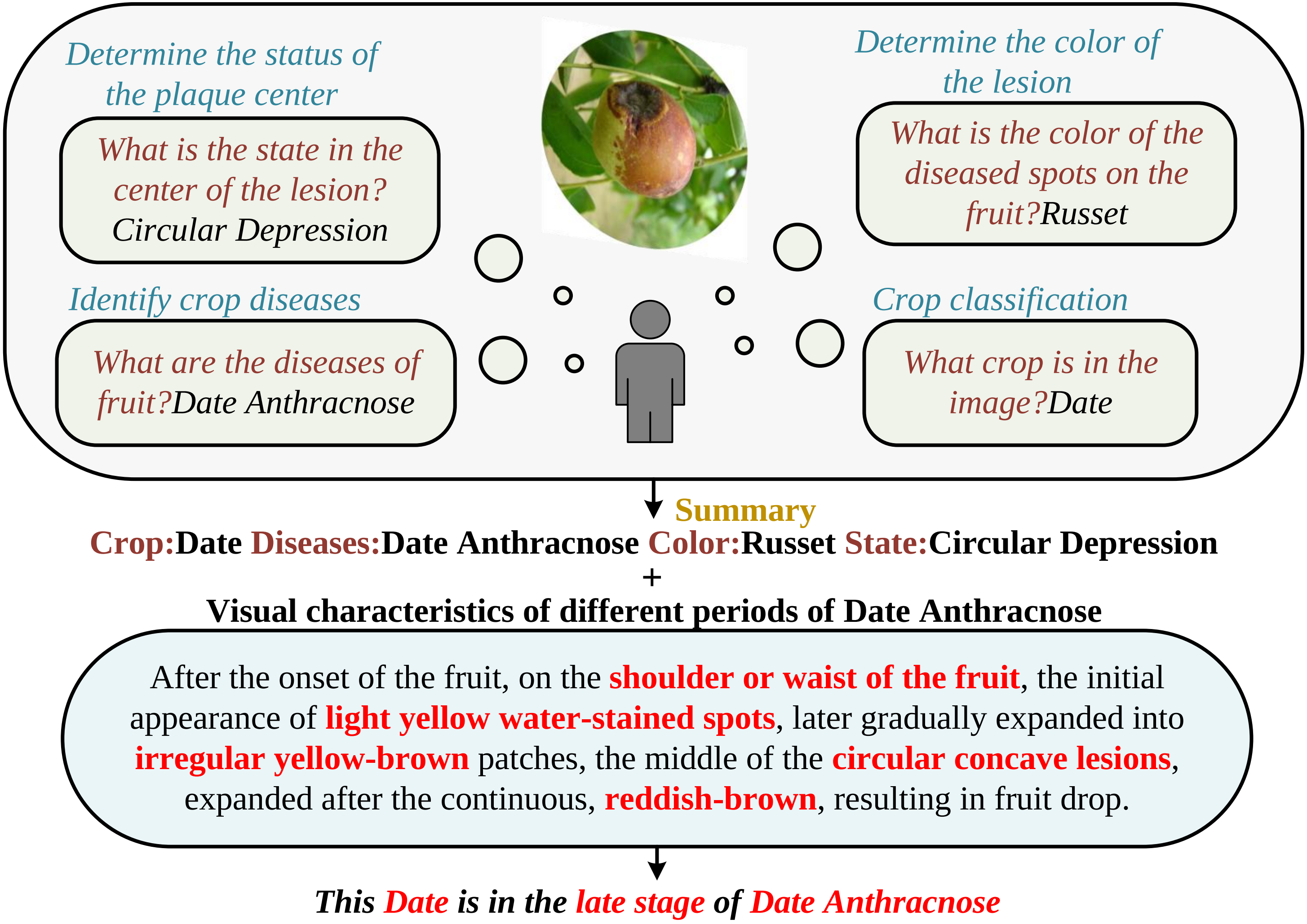

Determine the status of the plaque center
What is the state in the center of the lesion? Circular Depression
Determine the color of the lesion
What is the color of the diseased spots on the fruit?Russet
Identify crop diseases
What are the diseases of fruit?Date Anthracnose
Crop classification
What crop is in the image?Date
Summary
Crop:Date Diseases:Date Anthracnose Color:Russet State:Circular Depression
+
Visual characteristics of different periods of Date Anthracnose
After the onset of the fruit, on the shoulder or waist of the fruit, the initial appearance of light yellow water-stained spots, later gradually expanded into irregular yellow-brown patches, the middle of the circular concave lesions, expanded after the continuous, reddish-brown, resulting in fruit drop.
This Date is in the late stage of Date Anthracnose
