# Supplementary material for: Informed-Learning-Guided Visual Question Answering Model of Crop Disease
Source: Plant Phenomics. 2024 Dec 16;6:0277. doi: 10.34133/plantphenomics.0277 (PMC11649200; doi:10.34133/plantphenomics.0277)
Supplement: Supplementary 1 — Figs. S1 to S4 Tables S1 to S3 [file plantphenomics.0277.f1.zip › Fig-S2.pdf]

Disease category	Image	Questions	Answers
Strawberry anthracnose	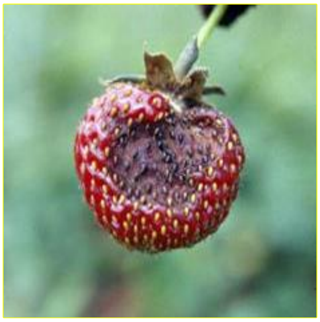	What is the fruit in the picture?<br>What color are the spots?<br>What is the texture of the plaque?<br>...	Strawberries<br>Russet<br>Rough<br>...
Tomato split fruit disease	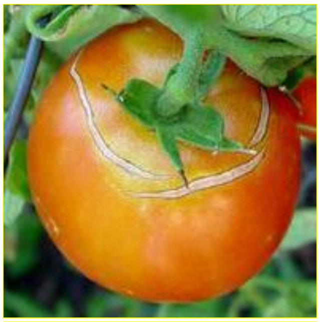	What is the type of fruit cracking?<br>What color is the center of the crack?<br>What color are the cracked edges?<br>...	Toroidal<br>Yellowish white<br>Tawny<br>...
Apple heart disease	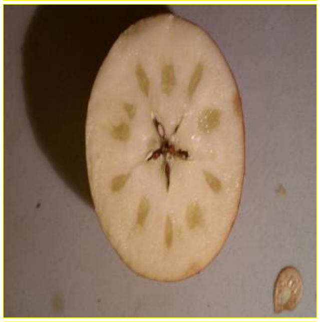	What shape is the plaque?<br>What color are the spots?<br>Are the edges of the spots clear?<br>...	Ellipse<br>Hazel<br>Yes<br>...
Pear rust	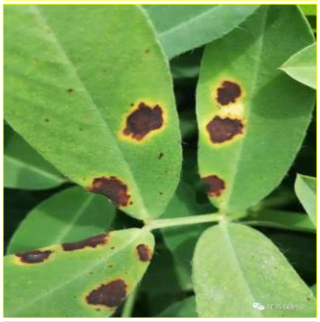	What is the number of spots?<br>What color is the edge of the spot?<br>What shape is the plaque?<br>...	9<br>Yellow<br>Frog-eye<br>...
Jujube anthracnose	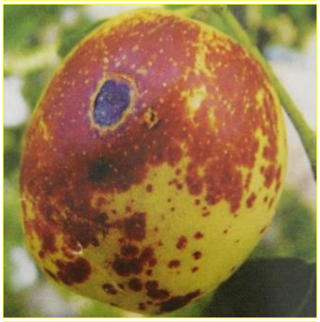	What is the fruit in the picture?<br>What is the status of the plaque center?<br>Where is the plaque on the fruit?<br>...	Jujube<br>Decay depression<br>Fruit kidney<br>...
Brown rot of pear	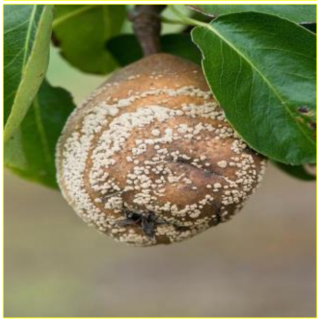	Are the fruit densely spotted?<br>What is the shape of the plaque?<br>What is the size of the plaque?<br>...	Yes<br>Graininess<br>Major spot<br>...
