# Supplementary material for: Informed-Learning-Guided Visual Question Answering Model of Crop Disease
Source: Plant Phenomics. 2024 Dec 16;6:0277. doi: 10.34133/plantphenomics.0277 (PMC11649200; doi:10.34133/plantphenomics.0277)
Supplement: Supplementary 1 — Figs. S1 to S4 Tables S1 to S3 [file plantphenomics.0277.f1.zip › Fig-S4.pdf]

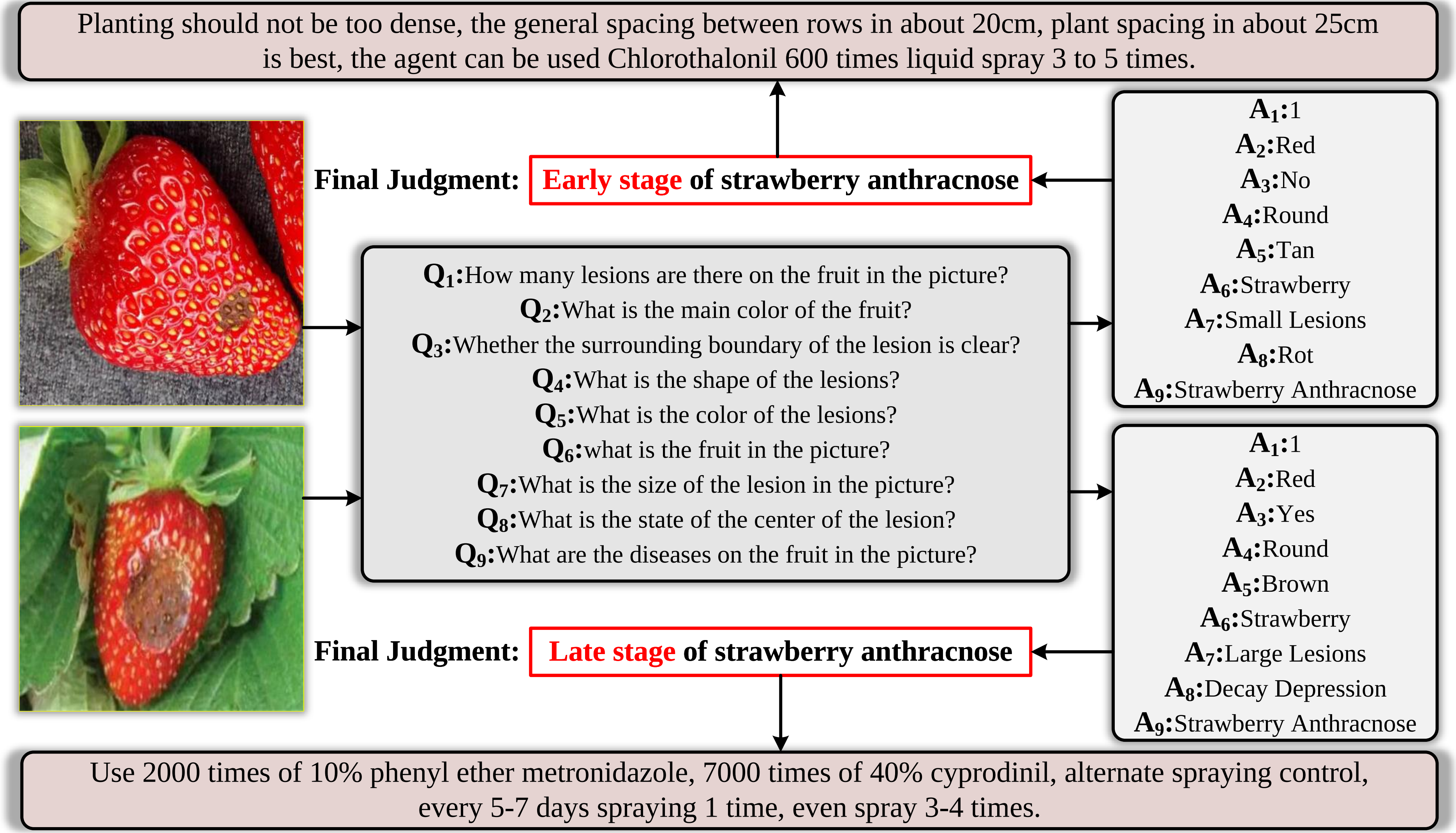
Planting should not be too dense, the general spacing between rows in about 20cm, plant spacing in about 25cm is best, the agent can be used Chlorothalonil 600 times liquid spray 3 to 5 times.
Final Judgment: Early stage of strawberry anthracnose
A_1:1
A_2:Red
A_3:No
A_4:Round
A_5:Tan
A_6:Strawberry
A_7:Small Lesions
A_8:Rot
A_9:Strawberry Anthracnose
Q_1:How many lesions are there on the fruit in the picture?
Q_2:What is the main color of the fruit?
Q_3:Whether the surrounding boundary of the lesion is clear?
Q_4:What is the shape of the lesions?
Q_5:What is the color of the lesions?
Q_6:what is the fruit in the picture?
Q_7:What is the size of the lesion in the picture?
Q_8:What is the state of the center of the lesion?
Q_9:What are the diseases on the fruit in the picture?
A_1:1
A_2:Red
A_3:Yes
A_4:Round
A_5:Brown
A_6:Strawberry
A_7:Large Lesions
A_8:Decay Depression
A_9:Strawberry Anthracnose
Final Judgment: Late stage of strawberry anthracnose
Use 2000 times of 10% phenyl ether metronidazole, 7000 times of 40% cyprodinil, alternate spraying control, every 5-7 days spraying 1 time, even spray 3-4 times.
