# Supplementary material for: Informed-Learning-Guided Visual Question Answering Model of Crop Disease
Source: Plant Phenomics. 2024 Dec 16;6:0277. doi: 10.34133/plantphenomics.0277 (PMC11649200; doi:10.34133/plantphenomics.0277)
Supplement: Supplementary 1 — Figs. S1 to S4 Tables S1 to S3 [file plantphenomics.0277.f1.zip › Supplementary Materials.docx]

# Supplementary Materials

*What crop is in the*

*image?*

*Date*

*What is the state in the*

*center of the*

*lesion?*

*Circular*

*D*

*epression*

*What is the color of the*

*diseased spots on the*

*fruit?*

*R*

*usset*

*Crop classification*

*What are the diseases of*

*fruit?*

*Date Anthracnose*

*Identify crop diseases*

*Determine the status of*

*the plaque center*

*Determine the color of*

*the lesion*


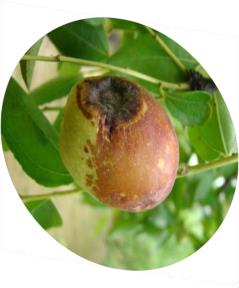


**Summary**

**Crop:Date Diseases:Date Anthracnose Color:Russet State:Circular Depression**

**+**

**Visual characteristics of different periods of Date Anthracnose**

After the onset of the fruit, on the **shoulder or waist of the fruit**, the initial appearance of **light yellow water-stained spots**, later gradually expanded into **irregular yellow-brown** patches, the middle of the **circular concave lesions**, expanded after the continuous, **reddish-brown**, resulting in fruit drop.

## *This Date is in the late stage of Date Anthracnose*

Fig. S1: Diagnostic process for crop disease stages. Leveraging the VQA task, acquire various visual attribute information concerning crop diseases to ascertain the precise stage of disease development depicted in the image, and devise treatment modalities tailored to various stages of crop diseases.

Disease Image Questions Answers


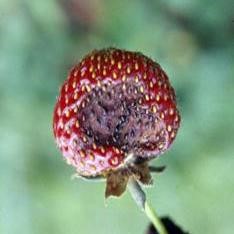

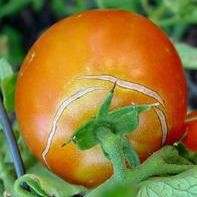

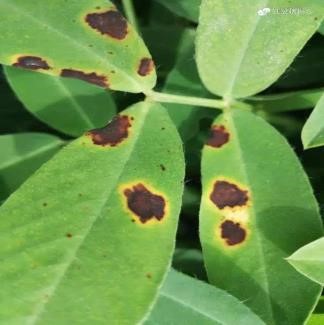

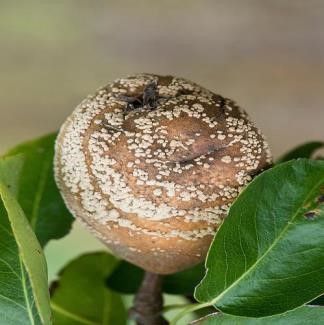

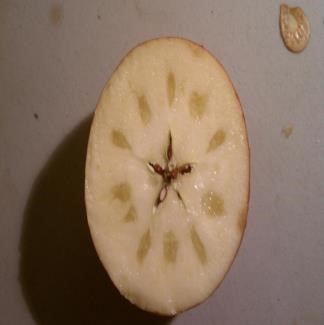

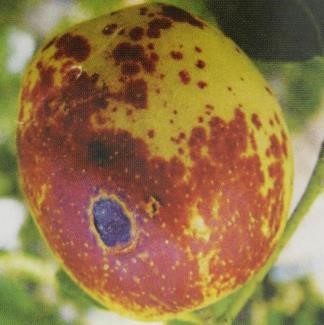


category

Strawberry

anthracnose

Tomato

split

fruit

disease

Apple

heart

disease

Pear

rust

Jujube

anthracnose

Brown

rot

of

pear

What is the fruit in the picture?

What color are the spots?

What is the texture of the plaque?

...

What is the type of fruit cracking?

What color is the center of the crack?

What color are the cracked edges?

...

What shape is the plaque?

What color are the spots?

Are the edges of the spots clear?

...

What is the number of spots?

What color is the edge of the spot?

What shape is the plaque?

...

What is the fruit in the picture?

What is the status of the plaque center?

Where is the plaque on the fruit?

...

Are the fruit densely spotted?

What is the shape of the plaque?

What is the size of the plaque?

...

Strawberries

R

usset

Rough

...

Toroidal

Yellowish white

Tawny

...

Ellipse

Hazel

Yes

...

9

Yellow

Frog

-

eye

...

Jujube

Decay depression

Fruit kidney

...

Yes

Graininess

Major spot

...

Fig. S2: Some examples of CDwPK-VQA. Each image is labeled with a series of questions, emphasizing fine-grained image features, curated by an expert guide to reflect the specific condition of the fruit.


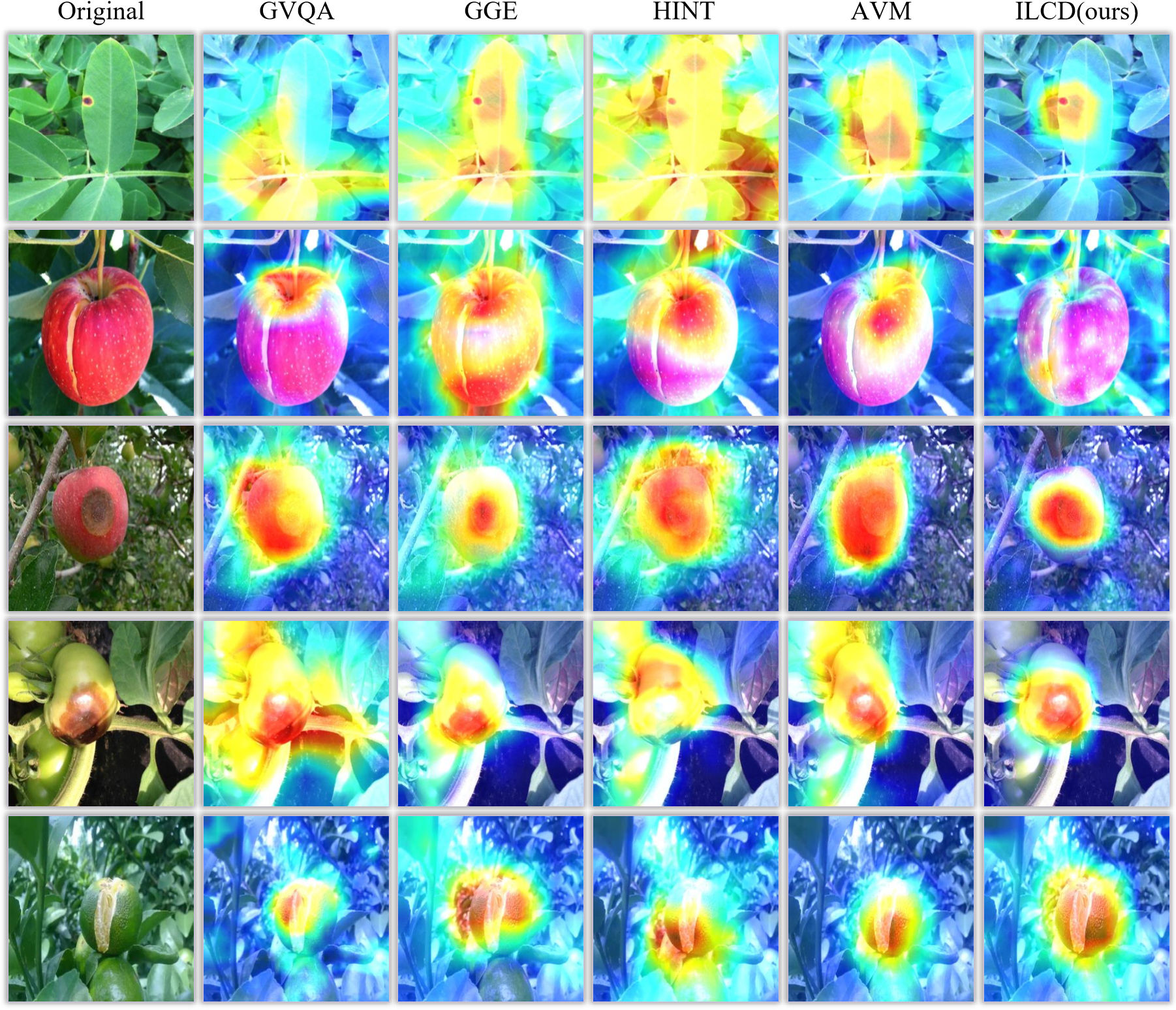


Fig. S3: Performance visualization comparison of five models. The question posed to the five models is “What is the color of the diseased spot in the picture?”. The models included in the comparison of attentional behaviors are “GVQA”, “GGE”, “HINT”, “AVM”, and “ILCD”. The dark red portion of the heat map represents the area where the models’ attentional behaviors are focused.


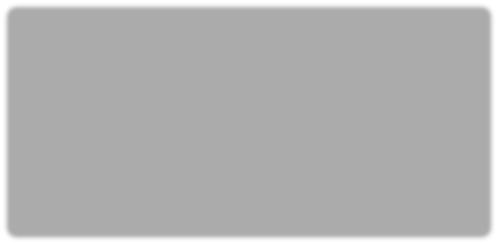


**Q**

**1**

**:**

How many lesions are there on the fruit in the picture?

**Q**

**2**

**:**

What is the main color of the fruit?

**Q**

**3**

**:**

Whether the surrounding boundary of the lesion is clear?

**Q**

**4**

**:**

What is the shape of the lesions?

**Q**

**5**

**:**

What is the color of the lesions?

**Q**

**6**

**:**

what is the fruit in the picture?

**Q**

**7**

**:**

What is the size of the lesion in the picture?

**Q**

**8**

**:**

What is the state of the center of the lesion?

**Q**

**9**

**:**

What are the diseases on the fruit in the picture?


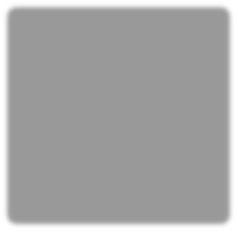


**A**

**1**

**:**

1

**A**

**2**

**:**

Red

**A**

**3**

**:**

No

**A**

**4**

**:**

Round

**A**

**5**

**:**

Tan

**A**

**6**

**:**

Strawberry

**A**

**7**

**:**

Small Lesions

**A**

**8**

**:**

Rot

**A**

**9**

**:**

Strawberry Anthracnose


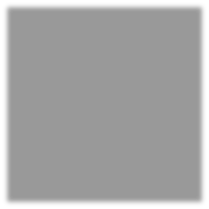

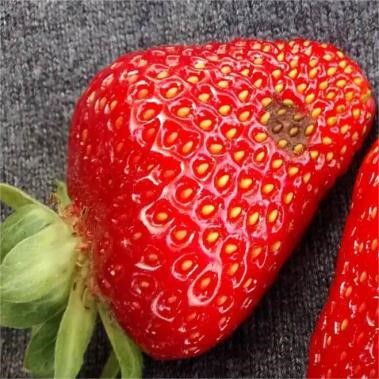

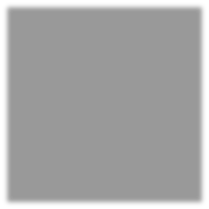

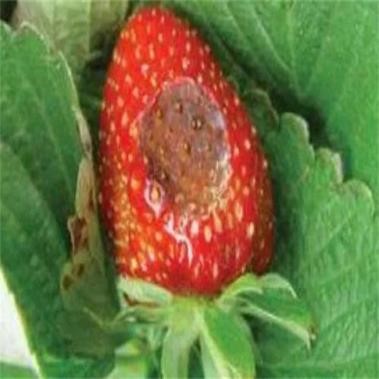

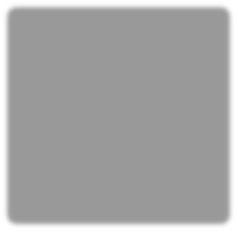


**A**

**1**

**:**

1

**A**

**2**

**:**

Red

**A**

**3**

**:**

Yes

**A**

**4**

**:**

Round

**A**

**5**

**:**

Brown

**A**

**6**

**:**

Strawberry

**A**

**7**

**:**

Large Lesions

**A**

**8**

**:**

Decay Depression

**A**

**9**

**:**

Strawberry Anthracnose

**Early stage**

**of strawberry anthracnose**

**F**

**inal**

**J**

**udgment**

**:**

**Late stage**

**of strawberry anthracnose**

**F**

**inal**

**J**

**udgment**

**:**


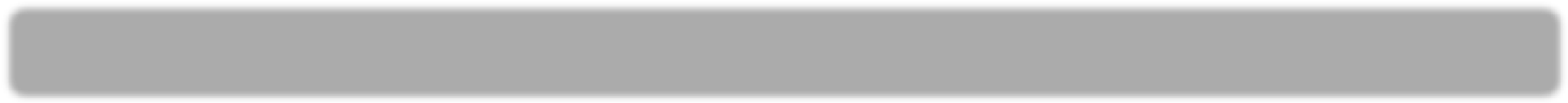


Use 2000 times of 10% phenyl ether metronidazole, 7000 times of 40% cyprodinil, alternate spraying control,

every 5

-

7

days spraying 1 time, even spray

3

-

times.

4


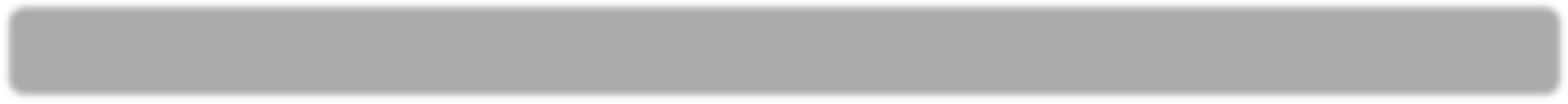


Planting should not be too dense, the general spacing between rows in about 20cm, plant spacing in about 25cm

is best, the agent can be used Chlorothalonil 600 times liquid spray 3 to 5 times.

Fig. S4: Inference of specific stages of crop disease development using VQA tasks. The ILCD is used to obtain answers to a series of questions posed, and these answers are used to reason about the specific period of occurrence of the disease contained in the images.

Table S1: Model parameters. Parameters of best quality of the model.

| Modality | Parameter | Value |
| --- | --- | --- |
| Image | Image Size | 448*448 |
|  | Output Dimensions | 1,024 |
| Text | Glove  LSTM | 300  1,024 |
|  | Max Length | 12 |
| Other | Batch Size  Learning Rate  Weight Decay  Dropout | 256  0.0001  0.2  0.2 |
|  | Optimizer | Adam |
|  | Activation | RELU |
|  | Attention Layer | 6 |
|  | Multi Head | 8 |

Table S2: The performance of using different image feature extractors. With “∗” indicating that no prior knowledge is involved in the training.

|  | Model | All | Yes/No | Number | Other |
| --- | --- | --- | --- | --- | --- |
| Coarse Grained | fbresnet | 79.46 | 95.87 | 75.22 | 77.46 |
|  | hsresnet | 80.04 | 96.04 | 73.15 | 78.57 |
| Fine Grained | Inception-v2  Vgg19  Resnet152 | 78.10  81.08  81.10 | 96.01  96.07  96.12 | 75.00  77.28  76.96 | 75.67  79.26  79.33 |
|  | Inception-v4^∗^ | 80.17 | 87.08 | 72.50 | 79.57 |
|  | Inception-v4 | **86.06** | **96.94** | **79.80** | **85.65** |

Table S3: Performance and model size comparison of three text encoders with different text lengths.

| Model Name | Text Length | All | Yes/No | Number | Other | Model Size |
| --- | --- | --- | --- | --- | --- | --- |
| ConvS2S  T5 | 5 | 70.13  82.95 | 84.69  93.74 | 71.11  78.58 | 69.72  81.49 | 547.31MB  8.95 GB |
| LSTM |  | 80.82 | 92.68 | 76.27 | 81.22 | 679.05MB |
| ConvS2S  T5 | 7 | 74.68  85.25 | 85.28  97.23 | 71.14  79.89 | 71.82  81.75 | 547.31MB  8.95 GB |
| LSTM |  | 84.37 | 95,83 | 79.62 | 84.16 | 679.05MB |
| ConvS2S  T5 | 12 | 76.24  85.87 | 86.34 **97.26** | 71.20 **79.86** | 73.03  82.74 | 547.31MB  8.95 GB |
| LSTM |  | **86.06** | 96.94 | 79.80 | **85.65** | 679.05MB |
