# Supplementary figures and images for: Informed-Learning-Guided Visual Question Answering Model of Crop Disease
Source: Plant Phenomics. 2024 Dec 16;6:0277. doi: 10.34133/plantphenomics.0277 (PMC11649200; doi:10.34133/plantphenomics.0277)

Original

GVQA

GGE

### HINT

A VM

ILCD(ours)

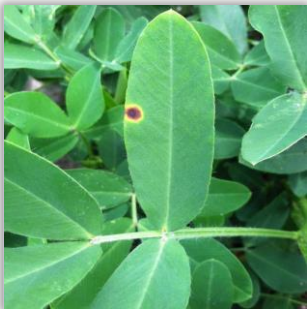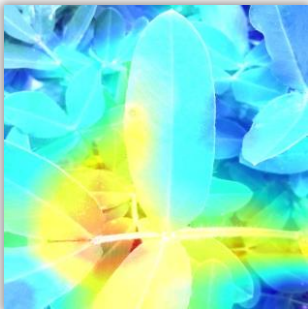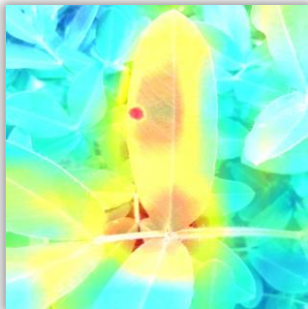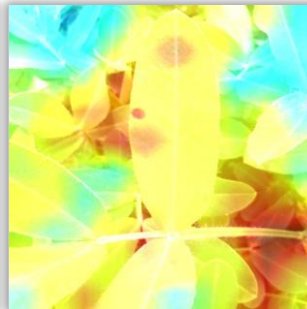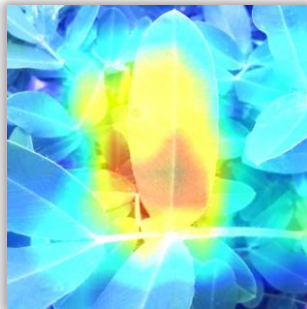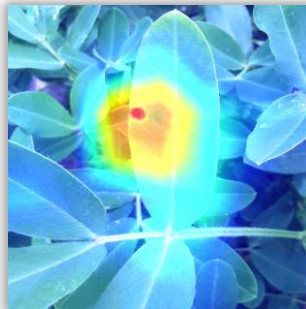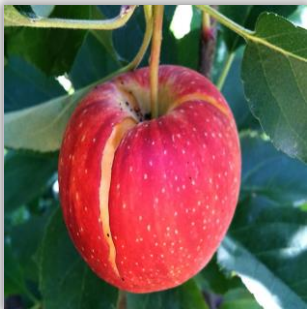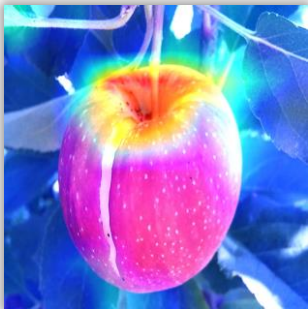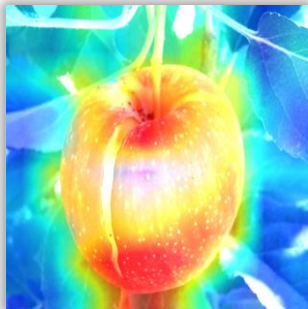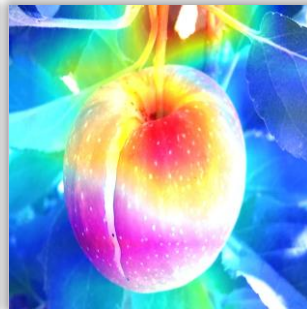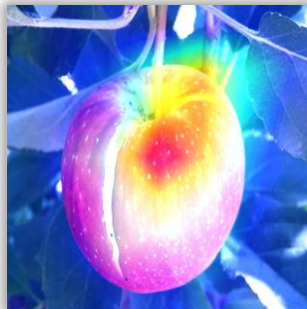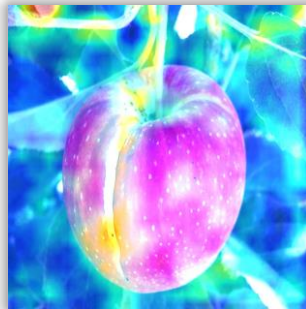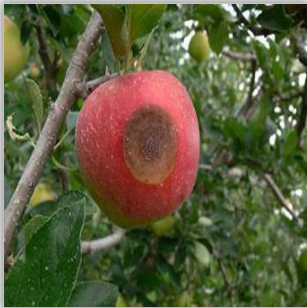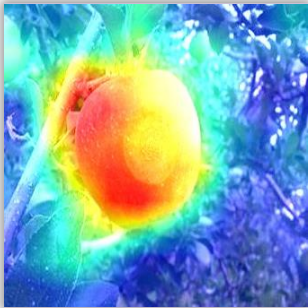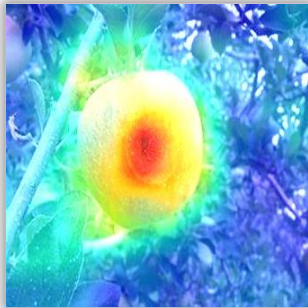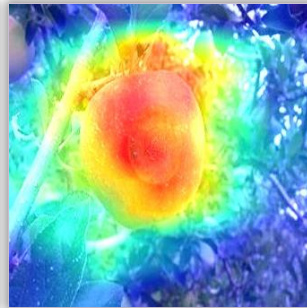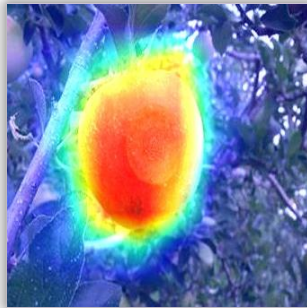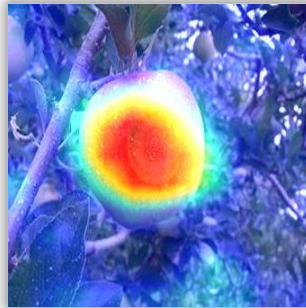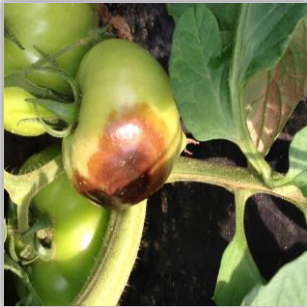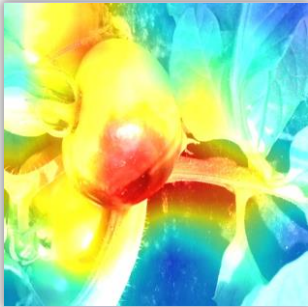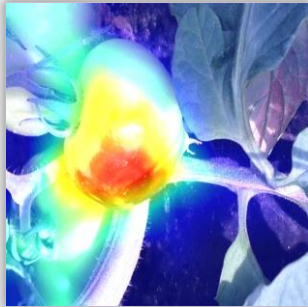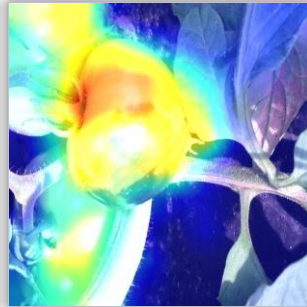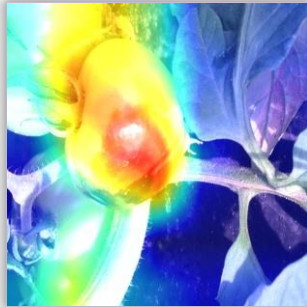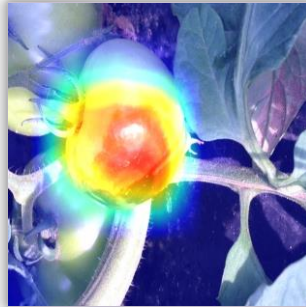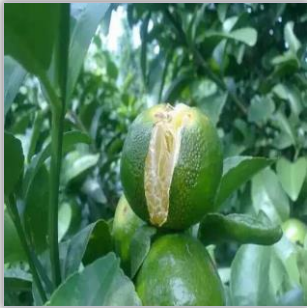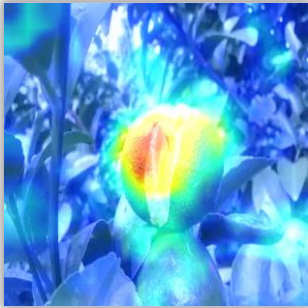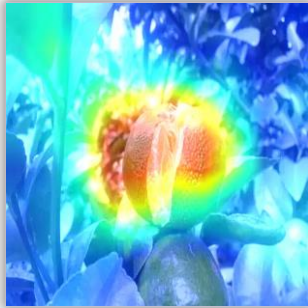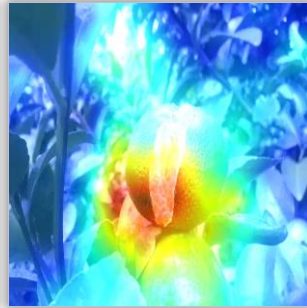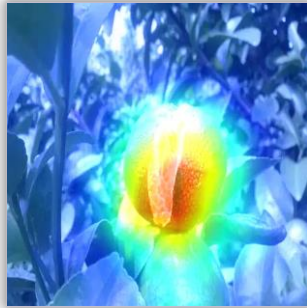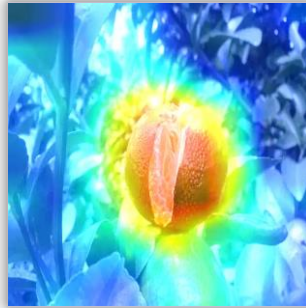

Supplement: Supplementary 1 — Figs. S1 to S4 Tables S1 to S3 [file plantphenomics.0277.f1.zip › Fig-S3.pdf]
